# “It’s taken away the most fundamental things about being human”: the lived experience and impact of post-SSRI sexual dysfunction

**DOI:** 10.1080/17482631.2026.2717470

**Published:** 2026-08-13

**Authors:** Seraj Brugi, Bob Budd, Craig Mackie, Sarah Fish

**Affiliations:** a Department of Clinical Psychology and Psychological Therapies, University of East Anglia, Norwich, United Kingdom; b Norfolk and Suffolk NHS Foundation Trust, Norfolk, United Kingdom

**Keywords:** Post-SSRI sexual dysfunction, PSSD, selective serotonin reuptake inhibitors, SSRIs, sexual side effects, lived experience

## Abstract

**Purpose:**

It has become increasingly recognized that sexual side effects from serotonergic antidepressants can last long after the medication has been discontinued, but little is known about the lived experience of post-SSRI sexual dysfunction (PSSD) and its impact on people’s identities, relationships, and mental health. This qualitative study aimed to understand the experiences of people living with PSSD in the United Kingdom.

**Methods:**

Ten participants were invited to take part in a semi-structured interview, and the data were analysed using an interpretive phenomenological approach.

**Results:**

Three themes, encompassing eight subthemes, were identified: (1) distrustful of professionals; (2) a changed me; and (3) living with the impact. The participants discussed their experiences of being dismissed and unheard, feeling emotionally numb, and making sense of the change and losses they have experienced. Implications for clinical practice include the importance of professionals being aware of PSSD and its potential impact.

**Conclusions:**

PSSD can affect every aspect of daily life, impacting more than just individuals’ sexual functioning and satisfaction. Further research is needed on effective treatments, including psychological interventions, which can offer meaningful support.

## Introduction

The medications used to treat common mental health problems are among the most widely prescribed drugs in the United Kingdom (UK), with the National Health Service’s (NHS) quarterly summary estimating that over seven million patients had been prescribed an antidepressant between October and December 2025 (NHS Business Services Authority, 2026). With the prevalence of common mental health problems continuing to increase since 2000 (McManus et al., 2016), it is no surprise that antidepressant prescription rates continue to rise in the UK population (Lalji et al., 2021), including a steady increase in children and adolescents (Dai Cao et al., 2021).

Psychotropic medications, including all serotonergic agents such as selective serotonin reuptake inhibitors (SSRIs) and serotonin and norepinephrine reuptake inhibitors (SNRIs), are known to have the potential to cause adverse reactions (Mayville, 2007). A meta-analysis comparing the efficacy and tolerability of SSRIs and tricyclic antidepressants found that whilst SSRIs were associated with better tolerability, experiencing adverse events was not uncommon (Anderson, 2000). Commonly reported side effects of serotonergic medication include sexual dysfunction, drowsiness, weight gain, dry mouth, and insomnia (Cascade et al., 2009).

Sexual dysfunction (SD) is an umbrella term that can refer to a variety of symptoms associated with a change in sexual experience, including loss of libido or desire, delayed or absent orgasms, and impotence (Jing & Straw-Wilson, 2016). It can be difficult to estimate the precise prevalence of SD amongst individuals using psychotropic medication; as many as half of patients experiencing sexual side effects may not discuss these with their healthcare providers (Rosenberg et al., 2003). Research has highlighted that embarrassment can be an important factor in preventing patients from discussing sexual concerns with their healthcare professionals (Akre et al., 2010; Gott & Hinchliff, 2003). Healthcare professionals are also likely to avoid this topic; a systematic review found that professionals in the UK are unlikely to ask about sexual functioning for various reasons, including discomfort and/or a lack of awareness of sexual health issues (Dyer & Das Nair, 2013). Mental health professionals are also often reluctant to query any sexual concerns with their clients, citing discomfort and, perhaps surprisingly, the belief that sexuality is a “peripheral issue” (Urry et al., 2019).

Moreover, research has demonstrated that SD—particularly a loss of desire and interest in sex—can be a common symptom of depression and anxiety in unmedicated individuals (Thakurta et al., 2012), leading some researchers to emphasize the difficulty in ascertaining a distinct role for psychotropic medication in inducing SD (Montgomery et al., 2002). That being said, it is clear from research findings that a significant number of patients experience sexual side effects following the use of SSRIs, with as many as almost three-quarters of individuals on some types of medication reporting concerns regarding SD (Montejo et al., 2001).

Whilst symptoms of SD are reported to usually resolve after the use of serotonergic medications has been discontinued, it has become increasingly accepted that this is not always the case. The incidence of post-SSRI sexual dysfunction (PSSD) has been reported to regulators since as early as 1991 (Healy, 2020), but the term was not coined until Bahrick (2006) identified reports of persistent SD following SSRI use in online peer support communities. Since then, there has been a growing body of research aiming to establish the biological plausibility, aetiology and presentation of PSSD, with differing theories on how factors such as gene expression, dopamine–serotonin interactions and hormonal changes may be responsible for the development of this condition (Bala et al., 2018; Peleg et al., 2022). No correlation between factors such as treatment length, substance use or somatic comorbidities and PSSD has been found (Linke et al., 2025). Overall, however, literature on the prevalence, impact, and effective management/prevention of PSSD continues to be scarce.

There are several challenges in estimating the prevalence of PSSD, with a recent systematic review (Tarchi et al., 2023) concluding that its frequency could not be reliably established from the current literature. Not unlike the difficulties in establishing the incidence of psychotropic-related sexual side effects, there may be challenges in making a differential diagnosis, potential underreporting of experiences, and potential dismissal by healthcare professionals. Moreover, it is unclear how frequently healthcare providers may ask about the experiences of PSSD when evaluating patients who discontinue their use of SSRIs. Healy et al. (2019) surveyed individuals from 23 countries on their experiences of speaking to healthcare professionals about PSSD, and found that patients often reported facing scepticism or invalidation when raising concerns about their symptoms, with some healthcare professionals denying the possibility that antidepressants could cause lasting changes in sexual functioning. Thus, whilst a recent retrospective cohort analysis estimated that the prevalence of PSSD may be 1 in 216 for individuals prescribed serotonergic agents (Ben-Sheetrit et al., 2023), underreporting by both patients and healthcare professionals is likely prevalent.

The impact of sexual side effects on quality of life can be significant. In a bid to understand coping strategies for the sexual side effects of ongoing SSRI use, O’Mullan et al. (2014) interviewed ten women in Australia under the age of 45 who were in heterosexual relationships. The analysis highlighted broad themes of suffering in silence, attempting to self-manage without medical support, and acceptance of the situation. When side effects persist after medication has been discontinued, the impact can be even greater; in a survey aiming to characterize people’s experiences of PSSD, the majority of individuals rated its impact on quality of life as very negative or extremely negative (Studt et al., 2021).

In a doctoral thesis, Stinson (2013) interviewed individuals with PSSD in America regarding the impact of persistent sexual side effects as well as the participants’ perceptions of what a psychologist may be able to offer. The participants described various consequences of PSSD, including an impact on relationships, identity and feelings about SSRIs. Similarly, Rice et al. (2025) conducted a study on the experiences of the prescription and cessation processes, as well as the perceptions and experiences of discussing the condition with healthcare providers, for participants with PSSD in Canada. The participants indicated a lack of understanding or acceptance of PSSD by healthcare professionals, leaving them to feel alienated and distrustful of medical services.

Whilst both studies provide valuable insight into the experiences of people with PSSD, there remains a need to expand on our understanding of this condition. To our knowledge, no study has attempted to explore the lived experience of PSSD for individuals in the UK. In line with the NHS’s national medicine optimization opportunities 2024/25 (NHS England, 2026), the NHS’s specialist pharmacy service (SPS) cautions against inappropriate use of antidepressants, highlighting the importance of shared decision-making and considering whether the harms of medication may outweigh the benefits for the patient (Specialist Pharmacy Service, 2026). Further research on PSSD will thus contribute to the literature by providing a better understanding of local patients’ experiences of taking antidepressants and what barriers may exist to achieving best practices.

Moreover, the lived impact of PSSD on individuals’ relationships with their mental health and their experiences with mental healthcare professionals has not been well-explored. Our study, therefore, aims to explore the impact that PSSD can have on people’s lives, mental health, identity and relationships, focusing on the lived experience of the condition for people in the UK.

## Materials and methods

### Ethical approval

This research was conducted in accordance with the Declaration of Helsinki, and the protocol was reviewed and approved by the University of East Anglia Faculty of Medicine and Health Sciences Research Ethics Subcommittee (ID ETH2425-0169). The participants provided free, informed, written consent for participation in this study and subsequent publication.

#### Ontology and epistemology

Adopting a position on the nature of reality (‘ontology’) and the nature of what we can know about it (‘epistemology’) is central to informing the theoretical underpinnings and the appropriate methodology for a research question (Al-Ababneh, 2020). For this study, a critical realist approach is taken, postulating that the world exists independently, but what we can know about it is constructed by and through our own lenses (Pilgrim, 2019). In line with this stance, an interpretive phenomenological methodology, rooted in critical realism and a hermeneutic epistemology, was adopted to give voice and interpretation to individuals’ lived experiences and perspectives in a participant-oriented manner (Alase, 2017).

#### Patient and public involvement

Embedding Patient & Public Involvement (PPI) in health research is essential to meeting people’s needs, helping to improve the quality of the research and what it can achieve for patients (NHS Health Research Authority, 2024). This study was co-produced with PPI, who helped shape the research topic and question to ensure that the aims were in line with the priorities of those directly impacted by the work. PPI contributions occurred throughout the study timeline; on the interview guide, to ensure that the questions were centred around lived experience, reviewing the recruitment strategy and materials to ensure that these were accessible to potential participants, and sense-checking the final analysis, as well as the organization of themes, to verify the veracity of our outcomes.

#### Design

This study adopted a qualitative approach to exploring the lived experience and impact of PSSD, using an interpretive phenomenological analysis (IPA) framework (Smith et al., 2022). The participants were invited to attend one semi-structured interview with the lead researcher (a male trainee clinical psychologist) lasting up to 90 min., with an interview guide used to prompt questions and further discussion.

#### Participants

The participants for this study were recruited via purposive and snowball sampling by liaising with and advertising via the UK PSSD Association, which aims to promote awareness and research into PSSD. The UK PSSD Association advertised this project on their website and social media. In accordance with the terminology and criteria used by the Association, participants who developed PSSD following SNRI use were also eligible for inclusion.

There are no specific criteria for determining sample size in IPA, with the emphasis being on data richness rather than quantity (Smith et al., 2022). This study aimed to recruit up to ten participants who met the following inclusion criteria:

1. Over 18 years of age;
2. Able to understand written and spoken English to a sufficient degree that would allow participation in an interview;
3. Self-identifies as experiencing sexual side effects that began after SSRI or SNRI use;
4. Sexual side effects persisted despite a period of cessation that lasted at least 3 months (as per the suggested diagnostic criteria proposed by Healy et al. (2022)).

#### Materials

Participants who expressed interest in the study were contacted and provided with an information sheet, consent form and an interview guide. Following appropriate consent, participants were invited to complete a brief demographic questionnaire including the questions below:

1. How old are you?
2. How would you describe your gender?
3. How would you describe your current relationship status?
4. When did you first use SSRIs/SNRIs? Please provide details of SSRIs/SNRIs you have been prescribed.
5. Did you experience sexual side effects which began after using SSRIs/SNRIs?
6. Have you had any gaps in using SSRIs/SNRIs? If so, how long?
7. Did the sexual side effects persist whilst you were not using SSRIs/SNRIs?

The interview schedule for this study was informed by Stinson's (2013) topic guide with amendments made by the research team in line with the aims of this study and PPI feedback:

1. Tell me about how you came to receive a prescription for antidepressants?
2. Tell me about what side effects you experienced when using antidepressants? Did you discuss any of these with a healthcare professional?
3. Tell me about how you came to realize that you were experiencing persistent, or ongoing, sexual side effects? What was that experience like?
4. How has post-SSRI/SNRI sexual dysfunction (or PSSD) impacted your life?
5. How has PSSD impacted your sense of self/identity? What is it like to be you?
6. How has PSSD impacted any relationships in your life? For example, how has PSSD impacted how you experience romantic relationships?
7. How has PSSD impacted your relationship with your mental health? What has that experience been like?
8. Thinking about what led to you receiving a prescription for antidepressants, tell me about how PSSD has impacted the way you cope with your mental health?
9. How has PSSD impacted how you feel about accessing support for your mental health? If you have accessed support since living with PSSD, how did you experience this?
10. Is there anything I haven’t asked about that you would like to mention?

#### Procedure

Participants were invited to take part in this study via a research poster that was advertised on the UK PSSD Association network and social media pages. The research poster provided information about the study, including the researcher’s contact details. Eligible participants who contacted the researcher to indicate their interest were provided an electronic information sheet, consent form, and the interview guide. The participants were given up to 1 week to review the forms and decide whether they wished to take part. The consenting participants were invited to complete a short questionnaire and to schedule an audio-recorded online interview at a mutually agreeable time that may last up to 90 min.

The interviews began with an introduction by the researcher, who reiterated the purpose of the interview and reminded the participants that they could withdraw or decline to answer a question at any time. After completion of the interview, the participants were provided with a debrief sheet thanking them for taking part, with signposting information for the participants to seek support should they wish to do so. The participants were electronically sent a £10 gift card for their participation. An automated transcript of the interview was produced via Microsoft Teams. The accuracy of the transcript was carefully checked; corrections were made whilst re-listening to the interview and any identifying information was anonymized.

#### Analysis

A diverse range of analytical methods can be utilized when completing an IPA study (Larkin & Thompson, 2012), and for this study, a six-step approach, adapted from (Smith et al., 2009, 2022) was undertaken to complete this analysis.

Firstly, the recorded interviews were listened to again, and the transcripts were re-read to allow the lead researcher to immerse himself in the data. Next, the researcher completed thorough note-taking throughout the transcript, with comments on the data being descriptive (describing the content of the interview), linguistic (considering the participant’s use of language), or conceptual (interpreting underlying concepts and subtext). Then, themes that emerged from the notes, referred to as personal experiential statements, were named, allowing the researcher to begin making sense of the participants’ experiences. Connections across statements were then identified; related statements that are relevant to the research question were re-organized and clustered into appropriate personal experiential themes (PETs) and subthemes. This process was then repeated for each individual case and, finally, patterns of convergence and divergence across all ten participants’ lists of PETs and subthemes were identified; related themes were then organized, appropriately renamed and clustered into group experiential themes (GETs) and subthemes.

An automated transcript was initially produced by Microsoft Teams, and accuracy was thoroughly checked when the researcher re-listened to the recordings. Exploratory noting and naming/clustering of experiential statements and PETs for each participant was completed using Microsoft Word (e.g., using lists), whilst identification of GETs across participants was then completed by hand (e.g., using printed lists and markers).

#### Reflexivity and quality assurance

Being aware of one’s own biases and preconceptions is particularly important when completing research that involves a significant interpretive element. Bracketing refers to the process by which researchers attempt to mitigate the influence of said preconceptions on the research process (Tufford & Newman, 2010). All the authors are mental health professionals who have worked with individuals taking SSRIs or other psychotropic medications in NHS settings. SB is a male trainee clinical psychologist with an interest in iatrogenic harm and clinical health psychology, having worked with many individuals experiencing chronic health problems impacting their mental health. SF is a clinical psychologist with previous experience in sexual health services and an interest in psychosexual wellbeing. As such, there will inherently be preconceived notions about the role of SSRIs, and associated sexual side effects, in mental healthcare.

Using a reflexive journal, which was maintained throughout the project by SB to reflect on thoughts and feelings that arose during data collection and analysis, can help aid learning and ensure the researcher is aware of their own biases (Vicary et al., 2016). Additionally, research supervision was utilized with all authors throughout the project to reflect on the process and emerging findings. Thus, whilst it may not be possible to completely remove oneself from the impact of our own biases, reflexive journaling and supervision played a significant role throughout the analysis to ensure a robust and high-quality study that can contribute meaningfully to the existing literature. Additionally, SF reviewed two of the annotated transcripts, providing feedback on the exploratory noting, the construction of experiential statements, and the labelling of PETs. The participants were also provided copies of their interview transcripts for comment if desired.

## Results

### Participant characteristics

Participant characteristics are presented in Table I, with minimum details reported to further protect anonymity within the local PSSD community. Interviews lasted from approximately 28 to 86 min. The age group and duration of PSSD are reported in 10- and 5-year ranges, respectively. Whilst the duration of PSSD refers to the length of time since the PSSD was first experienced, when taking into account the time spent on medication, some participants have experienced SSRI-related SD for longer than the range indicated. All participants described experiencing persistent genital numbness, a loss of interest in sex, or, in most cases, both (as well as other sexual side effects such as anorgasmia, erectile dysfunction, and vaginal dryness).

**Table I.** Participant characteristics.

Pseudonym	Gender	Age (years)	Duration of PSSD (years)
Alicia	Female	20–29	5–9
Arthur	Male	40–49	10–14
Bethany	Female	40–49	25–29
Frank	Male	50–59	<5
Jo	Female	40–49	10–14
Lucas	Male	30–39	<5
Michael	Male	30–39	<5
Stacey	Female	30–39	<5
Thomas	Male	20–29	5–9
Tony	Male	30–39	15–19

### Analysis

Three GETs comprising a total of eight subthemes emerged from the cross-analysis of participants’ transcripts: (1) distrustful of professionals; (2) a changed me; and (3) living with the impact. Table II illustrates participant representation across these themes as well as the related subthemes.

**Table II.** Representation of group experiential themes and subthemes across participants. ‘✓’ = theme was significantly represented in participant’s account, ‘O’ = theme was partially represented in participant’s account.

Theme	Alicia	Arthur	Bethany	Frank	Jo	Lucas	Michael	Stacey	Thomas	Tony
Distrustful of Professionals	✓	✓	✓	✓	✓	✓	✓	✓	✓	✓
*Not being informed*	✓	✓	✓	✓	✓	✓	✓	✓	✓	✓
*Dismissed and unheard*	✓	✓	✓	✓	✓		✓	✓	✓	✓
A Changed Me	✓	✓	✓	✓	✓	✓	✓	✓	✓	✓
*This emptiness inside me*	✓	✓	O	✓	O	O	✓	✓	✓	✓
*Struggling to relate*	✓	✓	✓	✓	✓	✓	✓	✓	✓	✓
*Who am I now?*	✓	✓	✓	✓	✓	O	✓	✓	✓	✓
Living With the Impact	✓	✓	✓	✓	✓	✓	✓	✓	✓	✓
*What PSSD has taken away*	✓	✓	✓	✓	✓	✓	✓	✓	✓	✓
*Taking PSSD to therapy*	✓	✓			✓	✓	O	✓	O	✓
*What helps me to cope*		✓	✓	O		✓	✓	✓	✓	✓

### Distrustful of professionals

This theme encompasses participants’ experiences of discussing persistent sexual side effects with medical and healthcare professionals, often resulting in participants feeling ignored and thus distrustful of healthcare. Two subthemes represent the various experiences: (1) not being informed and (2) dismissed and unheard.

#### Not being informed

All the participants described some sense of not feeling fully aware of the risks their medication potentially carried, with conversations around possible side effects (even whilst on the medication) being very brief or not taking place at all. For most participants, this conversation would have been relevant in the decision-making process; when asked how it felt to realize that the sexual side effects were not subsiding, Frank said “*Devastated. Yeah, devastated because I’d never, I’ve never experienced—I would have never touched them if I’d known there was any slight [chance] of what was going to happen”.* Perhaps Frank is *devastated* not just because of the impact of PSSD, but because, for him, the outcome could have been avoided.

Others also describe feeling uninformed of the risks, with some feeling intentionally misled. Arthur says it is “*heartbreaking”* that “*the clinical trials that proved SSRIs showed that PSSD was a thing and it was- it was covered up […] I mean, what are you supposed to think about that?”.* Arthur poses a question here, but his own answer is clear: *“All these people whose lives have been completely wrecked by these drugs, and we’ve been reporting it for years and nobody does anything […] I just don’t trust any of it”.* Arthur’s experiences appear to have made him distrustful of professionals, whom he can no longer rely on to be transparent. When talking about mental health and what led to antidepressants being prescribed, Michael expressed a similar sentiment, saying, “*I just feel like I’ve been scammed, basically. It’s like the mother of all scams”.* I wondered if the issue for Michael wasn’t that he simply didn’t know that PSSD was a risk, but that not knowing makes him, and others in his position, the victims of “*the mother of all scams”.*

The lack of awareness around PSSD meant that participants felt confused and shocked by their experiences, rupturing relationships with professionals—and the wider systems—that intended to support them. Whilst discussing her current medication, Alicia offered the following reflection:

*I’d say I’m a lot more wary of medications, really. So even now I’m kind of—I’m on Mirtazapine and I don’t even know whether that could be making things worse or not, but I think I’m really reluctant to kind of increase the dosage […] I feel, yeah, distrusting of… even doctors, really, I suppose in that sense. And yeah, medication for mental health. Yeah, just feel, yeah, distrusting.* (Alicia)

Alicia describes what sounded like an internal battle between accepting medication from her doctors and the *reluctance* she feels based on her past experiences. For most participants, it is understandable to blame the prescribers who, at best, had not heard of PSSD themselves or, at worst, did not disclose this risk. For Lucas, however, it became clear that he felt responsibility lies with himself; “*I’m not trying to seek to blame this [doctor], I put pressure on her, you know”,* later saying “*if I Googled it, maybe I would have found [PSSD], but I was naïve, you know?”* As Lucas requested medication and “*put pressure”* on his GP, I wondered if he felt responsible for making the decision to take SSRIs. Another possibly relevant factor here may be how prescribers respond when concerns around sexual dysfunction are raised; when mentioning PSSD, most participants felt their concerns were dismissed, whereas Lucas found his doctor to be “*kind of sympathetic, but I knew he wouldn’t be able to do much*”.

#### Dismissed and unheard

Participants frequently described feeling like they had not been heard, particularly in the context of healthcare appointments. This understandably leads to feelings of dismissal, rejection and neglect. After watching a British Broadcasting Corporation (BBC) documentary on SSRIs and possible withdrawal effects, Stacey raised her concerns with a psychiatrist:

*I was kind of a bit freaked out by that […] I was just like ‘shit’, like I don’t want to take this […] she [psychiatrist] was just like the BBC—she said to me, you know […] the BBC don’t know what they’re talking about.* (Stacey)

Whilst Stacey’s conversation with her psychiatrist wasn’t explicitly about PSSD, she says it reassured her that there was “*nothing new to worry about”*, something she would disagree with today after multiple difficult encounters which have minimized her experiences: “*you’re not really fully human. You feel less of a human. You feel completely dismissed”.* Here, Stacey alludes to the idea that to be human is to be heard and validated, meaning that she cannot be “*fully human”* when she is “*completely dismissed”.*

For Arthur, the fact dismissal came from within the healthcare system feels particularly difficult: “*you’re just basically being told that you’ve made it up and it’s- it’s the result of some, like, the kind of institutions that are kind of there to kind of care for you and help you, supposedly”.* Arthur’s use of the word “*supposedly”* seemed to suggest a mistrust of healthcare. Similarly, Tony felt exasperated by his experiences with various healthcare professionals: “*I got sick of health professionals not believing me about this condition, so I put in a complaint”.* He describes how this incident then led to further difficulties with the local crisis team that he had been in contact with at the time:

*I spoke to a woman [at the crisis team] and she was absolutely horrible. Like I mean, like really nasty. I started explaining to her how I was suicidal because of the sexual side effects that hadn’t gone away from the medication […] and she was really cold and unfriendly with me.* (Tony)

For Jo, the intersecting aspects of her identity as a woman accessing healthcare and as a patient seeking answers felt poignant for her:

*I think this is something maybe only women get: when I was [having counselling], they just kept telling me […] ‘oh, well, sexual dysfunction in women is very common’ […] made me feel like I’m some kind of weirdo for wondering where my sexuality went.* (Jo)

Jo was made to feel *weird* for asking questions about her sexuality, meaning that her concerns regarding PSSD were not recognized. Given their invalidating experiences, it is unsurprising that some participants sought answers elsewhere; Thomas had been searching online regarding his side effects when he came across PSSD: *“So I went into that rabbit hole and that’s when everything clicked”.* For Tony, after his GP said *“it can’t be the medication because it’s out of your system”,* he also turned to the internet: *“I started looking up online to see if there were other people who had suffered this, and I found other people”.* The need to feel heard and believed was salient in participants’ accounts. Dismissal—particularly from prescribers and health care professionals—betrayed their confidence and trust, adding to their distress and isolation.

In contrast, positive encounters with professionals could have a significant impact in helping participants feel seen. Describing a recent appointment, Thomas said: *“At the moment I’m seeing [practitioner at facility] and the woman on the phone straight away knew that it was PSSD. So, I’m really grateful for that”.* Despite only having a phone call and not starting any treatment yet, being instantly seen and heard, an experience new to him in the healthcare context, has made Thomas “*really grateful”.*

### A changed me

This theme encompasses how participants describe experiencing their own internal sense, including identity, and how this relates to their ability to connect with themselves and others around them. Three subthemes represent these experiences: (1) this emptiness inside me; (2) struggling to relate; and (3) who am I now?

#### This emptiness inside me

Many participants spoke at length regarding their experiences of emotional numbness following the use of SSRIs. In addition to persistent sexual side effects, Arthur said:

*My emotions were completely numbed. It was like I no longer could feel excited in anything or, you know, just emotional numbness. And it was like- I was in a relationship at that time. It was, you know, a really kind of nice relationship and it just kind of felt like I went from being in this position where I was really happy that I’d met this person and it was going really well […] like literally overnight, I kind of like couldn’t feel anything anymore for her.* (Arthur)

Arthur’s experience, which he describes as “*really horrible”*, is unfortunately not unique. When asked what emotions went through him when he realized his sexual side effects were not subsiding, Frank said “*That’s the problem, it takes away your emotions, you know? I’m walking around like a statue [chuckle]”.* Frank’s use of the word *statue* perhaps emphasizes to what extent he feels disconnected from any feeling and the world around him. Michael describes life now as “*like all the colour has gone”*, adding that “*it didn’t take much to make me happy before. Now, nothing makes me happy”.* This seemed to mean that the world now felt duller to Michael, who describes himself as previously being a “*really passionate person”.*

For Thomas, life has become so “*mechanical”* that “*there’s not a condition that I know about that I wouldn’t trade places with”.* The burden of emotional incapacity is so great that any alternative reality feels more bearable:

*For example, I have family members that have had cancer, and OK it’s like, it’s horrible. But you still have your emotions. If I could say I want my emotions for, like, I want to be with my emotions for five years and then after five years I can die. That’s the condition, let’s say. I will take that.* (Thomas)

Thomas’ statement that he would accept a condition of death if it meant that he could temporarily “*be with”* his emotions is striking. It appears that for him, a life without the feelings he once had is a fate worse than dying. A similar sentiment was expressed by Frank, who said:

*It’s [PSSD] almost like a living death sentence to be honest, I can’t think of anything worse. It’s even worse than cancer because at least with cancer, you know- OK you’re probably going to die with it, but at least you have your feelings intact.* (Frank)

Other participants reflected on the “*hollow”* or “*empty”* nature of emotional experiences with PSSD. Alicia reflected that relationships have been difficult, as she “*would go through things and come out kind of feeling kind of empty and, yeah, and just not kind of good enough”.* Similarly, Tony describes feeling “*empty”* during life experiences that he has had:

*They were completely hollow experiences where I couldn’t enjoy them or basically I couldn’t experience them. If I was to give you an analogy, it’s almost like eating chocolate when you’ve got a really bad cold or something. You can’t taste it, you can eat the chocolate but you can’t get any pleasure from it.* (Tony)

Alicia and Tony’s statements help to reconcile the idea that PSSD can cause complete emotional numbness for individuals, whilst said individuals are still able to reflect on the very difficult (e.g., *“really horrible”*) burden of the condition. Rather than complete apathy, a *“hollow”* and “*empty”* internal sense overshadows any capacity to experience other emotions. For Michael, he understands this as repressed feelings: “*I mean this [becoming emotional] is good. The fact I’m crying is good. I think I’ve got a lot of repressed emotion”.* Despite getting upset as he spoke about there being “*no escape”* from PSSD, Michael is relieved to see himself able to connect with his emotions. There is no doubt, however, from participants’ accounts that the ability to experience and engage with positive affect, or indeed any affect, has been significantly affected by SSRIs.

#### Struggling to relate

All participants described an impact on relationships with others, whether that be romantic relationships, relationships with friends and/or families, or being able to relate to peers. Stacey said “*this condition really does, like, isolate you, because you can’t relate. You cannot relate to people”.* Arthur expressed a similar sentiment, saying that PSSD is “*very isolating […] when you’re in this position where it’s like you’ve lost your connection to life”.* The level of difference and disconnection “*from life”* instilled by PSSD seemed to have isolated Stacey and Arthur from the world around them.

For some, because of this difference and disconnection, society itself can become a trigger. Tony said, “*there’s also a lot of triggers within friendship groups with people who don’t have PSSD when they talk about sex, dating, you know? […] you have to be a bit like an actor”.* What Tony describes, given that he now finds little in common with old friends, sounded like a performance. Confiding in friends and family can also be a difficult experience; Frank says that he only told one friend that he had “*no libido*”:

*He [friend] said “oh, yeah but most men our age lose a bit of their libido”. But he doesn’t understand that he might have lost a little bit of his libido becausemost men over 50 do, but [laughs] I’ve lost all of it, so it’s a different aspect to what he’s referring to.* (Frank)

Frank laughs, perhaps in exasperation, at a friend’s attempt to relate to his experience of PSSD, emphasising the fact that for Frank, what he experiences does not feel like ‘normal ageing’.

Whilst most participants were single at the time of the interview, some talked about the impact of PSSD on long-term relationships. Jo described how the condition led to her questioning a relationship she had been in for several years:

*It never occurred to me that six months after I was off [medication], 12 months after I was off it, it never occurred to me that it was still the drug at this point. I was thinking [it] must be, I don’t know, maybe it’s a relationship issue, maybe it’s me and my partner? […] It must be me, or it must be us, it must be him. It must be something going on*. (Jo)

Jo’s relationship survived these questions, but others spoke about attempting to date without success. Thomas spoke about a relationship that ended after his sexual side effects impacted his partner’s self-confidence: “*she became self-conscious because she thought it was her […] She was like ‘oh, well with the numbness, like do you not feel pleasure?’ And this is like- I don’t feel pleasure. But I can’t say that”.* Thomas was mindful of his partner’s experience in their relationship and describes feeling that he “*can’t say”* what he was truly thinking, which may have added to his shame around PSSD. He says that the “*pressure” to* hide the extent of his sexual side effects means that he is “*very hesitant in trying to go into something else [relationship]”.*

Two female participants spoke about how PSSD has increased their vulnerability in the context of relationships, leading to sexual encounters that they found unsafe or demeaning. After talking about how it can be “*really, really challenging”* to bring up PSSD when dating, Alicia said:

*There’s been times where I’ve kind of - after sex, I’ve sort of like, been in tears. Like I’ve been crying and almost felt a bit like, sometimes I think I’ve almost felt a bit used as well at times. Because I’ve kind of gone along with things that sometimes I haven’t been happy about just to please that person*. (Alicia)

Alicia’s understandable reluctance to discuss her sexual side effects with prospective partners, combined with the fact that she was not able to enjoy these experiences herself, meant that it was more difficult to talk about sex and boundaries, which perhaps contributed to her being vulnerable to feeling *“used”*. Bethany also recounted an unsettling experience with a “*really toxic”* partner:

*The only way I can describe it now is like he was sexually abusing me […] If I haven’t had this [PSSD], I wouldn’t have even tolerated him for 5 min. […] he said to me something that I didn’t really understand at the time, and like it’s only now since I’ve realised that I’m actually numb there [genitals] - basically what he said was he liked to be with me because, like, I guess I just didn’t react [due to numbness], and like, so he could abuse me in a way*. (Bethany)

Alicia’s and Bethany’s difficult experiences highlight the scope and magnitude of impact that PSSD can have on relationships; in their case, the capacity to understand and engage in safe sexual relations was compromised by their dysfunction. For Bethany, who is now able to make sense of her experiences, PSSD appeared to play a significant role in keeping her “*trapped”* in a dynamic that was “*highly toxic and highly abusive”.*

Partnerships can thus look very different from PSSD, leading some to think about how they can find new ways of relating and finding fulfilment in connecting with others. When reflecting on his current relationship, Lucas said:

*I’m probably on the more optimistic side in terms of I would generally believe that one can always find someone […] I think relationships are always possible for anyone, you know? But it’s just that there’s a million roadblocks in the way for people with [PSSD], and it’s going to be a relationship that’s much more negotiated and much more based on compromise and practicalities, frankly, you know? More of a, maybe more of a functional than a romantic relationship.* (Lucas)

#### Who am I now?

In light of the physical, emotional and relational impact of PSSD described so far, it is unsurprising that most participants spoke about how PSSD forced a fundamental change in what it meant to be themselves. Thomas reflected that “*in terms of identity […] I’m having to remember who I was to try and still be that person”.* It seemed that for Thomas, to preserve at least some sense of self, life has become a script. Similarly, when asked if he is the same person as before, Frank said *“I’m different. I’m totally different. It’s not me. Every morning I’m looking in the mirror, I’m thinking it’s just… this living nightmare continues”*. Frank does not say what he sees when he looks in the mirror, but I wondered if it was not the person he once was. Arthur says “*there’s all these aspects to my personality which are just not there anymore. It just feels like I’ve kind of got brain damage and just been like, yeah, just like kind of lobotomised”.* Arthur’s PSSD has not only changed his personality, but it has “*lobotomised”* him; the use of this term, a controversial medical procedure, alludes to the extent of the medical harm Arthur feels he has experienced.

The nature of PSSD means that it can have a significant impact on core aspects of self-identity, such as sexuality. Stacey described how her relationship with her sexuality has been forcefully altered by her experience:

*I don’t really consider myself asexual either because this is- No, I’m not asexual. But it’s almost like they’re forcing- I’m forced to be asexual in a way, if that makes sense. Because I never, you know, I don’t identify like that, but the condition has made me feel like that*. (Stacey)

Stacey’s statement conveys the incongruence between who she feels she actually is and who she has been “*forced”* to become, with her initial statement of “*they’re forcing-”* implying that someone or something is to blame for what is an unwelcome conflict in self-identity. For Michael, who had been unsure of his sexual orientation before being prescribed SSRIs, the sexual side effects leave him with an unresolved question regarding his identity:

*To have your sexuality blunted while you’re trying to figure it out is like, makes things really, like, difficult. So I don’t even bother now. I’m just, well, like I’m castrated now, so it doesn’t really matter what I am […] there would have been a lot of other things I could have done to figure [my sexuality] out.* (Michael)

Some participants also described how PSSD impacts their sense of self in relation to gender and social norms. When asked why she felt PSSD had affected her self-esteem, Alicia explained that she feels “*less womanly, I suppose […] I feel out of touch with my sexuality a bit, yeah”.* Expressing a similar sentiment, Stacey said that “m*y sense of self as a woman and a sexual being has been stripped away from me”.* For Alicia and Stacey, to be disconnected from their sexuality as women meant that their sense of womanhood had been altered.

Male participants described similar experiences; Tony said “*I don’t know if you can remember back to when before you went through puberty […] that’s what it feels like having PSSD. You feel like a prepubescent child”.* In this statement, Tony conveys a sense of being a ‘boy’ rather than a ‘man’, an experience echoed by Michael, who said “*it just affects - it’s like the core of your manhood […] it’s like got me right in the centre and everything proliferates from that”.* PSSD fundamentally alters identity and sense of self in a way that, for Michael (and others), means its impact “*proliferates”* into every aspect of life.

### Living with the impact

This theme encompasses how participants experience the impact of PSSD on the lives they envisaged for themselves, and how they begin to make sense of this impact. Three subthemes represent these experiences: (1) what PSSD has taken away; (2) taking PSSD to therapy; and (3) what helps me to cope.

#### What PSSD has taken away

Participants frequently talked about the life and future they felt they had lost as a result of PSSD. Michael feels that SSRIs *“totally tear someone’s life to shreds”,* expressing his regret at the decision to take the medication:

*Everyday I’m just like, why did you do that [take SSRIs]? That was so stupid […] There’s just a million things I would have loved to have done, and now I’m basically just paralysed because there is no quality of life when you’ve got this condition. It’s just shit*. (Michael)

Michael describes what feels like an altered course of life, leading to the loss of opportunities that were meaningful to him. Similarly, Lucas spoke about what PSSD has taken away from him: “*it’s completely transformed my life on every measure from my day-to-day life […] to my spiritual, mental world - interior world - to my expectations for the future”,* later adding that “*I do believe it’s maybe possible to establish some sort of life with this […] it’s not going to be the life you ever thought you would lead”.* Whilst a meaningful life is absolutely possible with PSSD, Lucas reflects sincerely on the fact that this life is different from the one he expected to lead, requiring an enormous shift in perspective.

After “*seeing the impact over my life”,* including what she had discussed regarding its impact on relationships, Bethany described what she felt PSSD had taken from her:

*It’s taken away the most fundamental things about being human […] about my experience to connect with another person emotionally, physically, sexually, it’s like—it’s probably taken away the right for me to be able to have a normal family life and have children with somebody else. I mean that’s what you—that’s what life is about, and I feel like that’s been taken away from me.* (Bethany)

For Bethany, PSSD has “*tentacles”* that influence various aspects of life and its trajectory. Her use of the word *“tentacles”* perhaps speaks to the wide-reaching and forceful hold that PSSD has in her life. Similarly, when describing its impact on his life, Thomas articulates a sense of being engulfed by the condition:

*PSSD reminds me of like a sphere. Like, it’s so perfect in how it like—not perfect in a good way, but how it encompasses the whole system with like your sexual functioning, your emotions, it’s just like this, yeah, like, perfect in how it affects everything.* (Thomas)

Tony said that even if he “*was to recover”* in the future, there would be no way of living the experiences he could have had in the past: “*I feel like the sort of experiences that I missed out on or that I couldn’t enjoy […] I can’t really replicate them in the same way”*, reflecting that experiences such as “*dating, sex life, [and] travelling as a young person”* would feel different should he have these opportunities later in life. It seemed that PSSD had a profound impact on participants’ hopes and expectations from life whilst simultaneously shaping new narratives, as reflected by Jo, who said: “*it’s what [PSSD] has removed, then it’s also what it’s forced me to become”.*

#### Taking PSSD to therapy

Given the impact of PSSD on every aspect of individuals’ lives, it is unsurprising that several participants discussed their experiences of seeking counselling or talking therapy in a bid to feel supported and validated. For Lucas, going to counselling—even if briefly—was helpful:

*I found it useful with PSSD, you know, the six sessions I went to, I- you know, that was good. It was useful. I struggle to think how anyone couldn’t find that useful in any situation, like being able to talk about your problem with someone who’s listening*. (Lucas)

Arthur found cognitive behavioural therapy (CBT) helped him “*with the understanding […] the experiences in my sort of past that had caused me to kind of have these sort of recurring problems with anxiety and depression”,* saying that PSSD *“pushed me to have to understand all the different factors that kind of go into, you know, keeping it kind of well mentally”.* Having someone listen to what you are experiencing whilst also helping to create a shared understanding of your story seems to thus be validating, perhaps especially if one has found little validation in other interactions and relationships, or where the veracity of one’s experiences has been questioned.

Not all participants, however, described positive experiences with mental health professionals. When raising PSSD within a mental health service, Jo says that her psychologist responded with “*I’ve heard of it, but it’s not official, you know, I can’t talk about this”.* When thinking about how her experience could have been improved, Jo reflected that “*she [psychologist] could have wrote back to my GP and said this needs investigation, but she didn’t”*. Stacey spoke at length about her experiences in therapy, saying she attended sessions with a mix of different mental health professionals. When reflecting on why she felt these sessions were “*useless”,* Stacey said:

*Because what happens is it ends up me having to educate them on my experience. I have to coach them […] to understand what’s going on, and what I’m going through. And like, you know, the whole point of, you know, counselling is for them to help me understand what’s going on, like going through my challenges, whereas I felt like it was just more about informing them.* (Stacey)

Educating her therapists on what PSSD meant was not the role Stacey wanted to assume in sessions. Conversations around the condition and its aetiology can invite questions that might be experienced as suspicious or invalidating. When asked what comes to her mind when she thinks about accessing therapy now, Stacey said, “*not denying your experiences is really important […] I don’t want any of those kind of questions that allude to ‘are you sure?’”.* For Stacey, and likely others, perhaps the issue is not that the therapist is asking questions, but that the question being posed here denotes that the therapist is not *sure* themselves if PSSD is ‘real’. A nuance illustrated by a conversation with Alicia, who, when asked what she would hope from a therapist, said “*for them to know that [PSSD] does exist […] I suppose just for them to kind of be there and listen and just listen to people’s experiences, and get an insight into how- what it’s like to have the condition”.*

#### What helps me to cope

Several participants spoke about how they manage the impact of this debilitating condition. Arthur framed PSSD as “*like being in a horror movie that you can’t escape from. That’s the only way I can really describe it. But, you know, I’ve had to learn to kind of, to survive, you know?”.* For him, in addition to having CBT sessions, this means “*managing”* what he can for his mental health:

*I have focused on my diet a lot, which I think has a massive difference, so I don’t drink alcohol anymore since I got PSSD. I don’t drink caffeine anymore since I got PSSD, and also I try to really limit the amount of sugar that I consume […] and those things kind of limit the kind of up and down in my mood […] I’ve always done a lot of exercise, you know? […] I do a minimum sort of amount of exercise every day*. (Arthur)

After reflecting that the *“loss”* of his sexuality changed his “*conception of [himself]”*, Lucas says he “*started going to church, which is a bit of a Hail Mary move to be honest […] but when you’re in the trenches, it’s like you’re clutching for something”.* The fundamental change in identity has led Lucas to search for something deeper to offer some fulfilment. Later, he described “*positive coping mechanisms”* that have helped him:

*I did read a bit of a book on Stoicism really early on in this [PSSD journey], and that gave me a few okay habits in terms of like, being really easy on myself, having generally low expectations and looking at any small win as a huge success. Not looking at the past, you know, we’ve talked a lot about the past today, but I don’t generally beat myself up about it. I just, like, in Stoicism they would say there’s nothing you can do. It’s happened.* (Lucas)

When asked about her relationship with her mental health, Stacey said, “*distractions”* are how she copes: “*I try to have a routine, I guess. I try to exercise as a distraction. Having a routine is good for distractions. It’s just, I guess it’s better than laying in bed all day”.* Being able to do this, however, took time for her: “*the first year [of having PSSD], I just could not get out of bed”*. It understandably took Stacey some time to process the changes she had experienced. Tony also spoke of the time it took him to begin to accept the reality of what had occurred:

*The first three years of having PSSD I was very suicidal and I didn’t cope with it well […] but one thing I would say is having PSSD for a very long time, like [15-19] years, it’s kind of built this sort of mental toughness […] I can deal with it a lot better now*. (Tony)

Tony also spoke of brief “*windows”* where he notices “*improvements”* in sexual functioning: “*My sexuality occasionally comes back - not completely to how it was before - but partially returns and then it just vanishes again. And it’s like Heaven”.* Tony’s use of the word “*Heaven”* seems to highlight the extent to which PSSD can feel like ‘Hell’. Some other participants also spoke of these brief glimmers of hope; Bethany said that she spent some time “*trying to do things”* to improve the dysfunction she was experiencing:

*I started fasting and I started going in the sea and I noticed these little windows, and - when I was eating really well, like, and not eating any sugar or processed foods […] I was getting little windows of like ‘ooh’ […] I felt joy for the first time […] and I noticed, like, my libido kind of picked up a little bit.* (Bethany)

Michael spoke of “*tiny little moments where I feel something”,* which are what keep him going: “*sometimes I’ll get a little bit of pleasure out of listening to music still. It’s nothing like it used to be, so like really, I’m—that’s what I live for now”.* For Frank, who said he experienced a “*little window”* of improved sexual functioning recently before “*[crashing] again”,* the momentary change brought joy but also understandable distress as it dissipated: “*it’s terrible [losing that window], I actually- because when I got the window, I was crying. I actually started crying with joy because I thought ‘oh, I’m feeling better!’, but it was just a brief window”.* This brief window was a reminder of what Frank had lost and what he could still have, making it a bittersweet experience.

Given the fact that almost all participants expressed a sense of feeling isolated and dismissed, it is unsurprising that finding validation and support within the PSSD community can be important. After speaking about individuals with PSSD supporting each other with various tasks, Michael said, “*There’s a lot of, like, skills in the PSSD community that sort of, you know—it’s something we’ve got that we can still help with”.* There is significant value in connecting with people from the community who can offer peer support and validation, as reflected by Tony, who said:

*I do find I’ve got some support from the PSSD community. I don’t know if you’re aware of the PSSD Network? And they’ve been very supportive and that has helped […] they’re the only people that I really- can really understand me.* (Tony)

## Discussion

This study aimed to further our understanding of the lived experience and impact of PSSD for participants in the UK, with a focus on deepening our knowledge of how the condition affects people’s mental health and their experiences of mental healthcare. An IPA approach identified three group experiential themes across ten completed interviews: (1) distrustful of professionals; (2) a changed me; and (3) living with the impact.

Participants frequently spoke about how they felt uninformed regarding the possible risks of antidepressant use, as well as conversations with healthcare professionals who often felt dismissive and invalidating. No participants stated that they were aware of PSSD before commencing medication, and all individuals described having no or very brief conversations about possible side effects. Our results are consistent with findings from Healy et al. (2019), Rice et al. (2025), and Stinson (2013), whose participants also described often feeling uninformed, ignored or devalued by healthcare providers involved in their care.

Several participants discussed their experiences of therapy, whilst some reflected on what they might hope and expect from a mental health professional should they engage in that work. Experiences differ widely between individuals; Lucas and Arthur, for example, found counselling and CBT valuable, respectively. For Stacey, however, having to educate therapists on PSSD meant that the sessions did not feel helpful. A common theme across conversations with participants highlighted the importance of feeling seen and believed by the professional. Research has shown that validation is central to fostering a sense of belonging and reducing negative affect (Benitez et al., 2019; Kim & Kim, 2013), and as many individuals with PSSD receive little validation in their experiences with professionals, it is understandable that some may feel reluctant to engage in therapy, or find that their experiences deepen their sense of invalidation.

Participants frequently spoke of feeling emotionally numb or blunted, describing their experiences of diminished positive and negative affect in various contexts. For some, whilst PSSD refers to persistent sexual side effects, it is fundamentally underpinned by a disconnection from one’s previous emotional capacity, a disconnection that will inevitably also have an impact on sexual functioning. Emotional blunting has been widely reported in the literature as a potential side effect of antidepressants; however, there is some disagreement as to whether patients’ experiences reflect a property of the antidepressants or a symptom of depression (Jawad et al., 2023). For participants with PSSD, however, it is clear that antidepressants have had a profound impact on their experiences of emotion, an impact that has persisted long after the medication. Moreover, PPI was instrumental in helping us make sense of the apparent contradiction between feeling emotionally “numb” whilst being able to reflect on the negative feelings that result from living with PSSD, noting how a consuming sense of internal hollowness can make it very difficult to feel or engage with any positive affect.

Most participants discussed at length the consequences of persistent sexual and emotional side effects on their identity and relationships, a finding that has been noted in previous research (Rice et al., 2025; Stinson, 2013). However, the present study goes beyond this to highlight an increased vulnerability to problematic intimate relationship dynamics for some individuals as a result of PSSD. Alicia recounted her experiences of sometimes “[feeling] used” as she had been reluctant to name or discuss PSSD with partners, making conversations around sexual boundaries and desires difficult. Bethany recounted “highly abusive” experiences, which she attributes both to her physical experience of genital numbness and a lack of understanding of what constitutes a “normal” relationship. This ultimately created an unequal sexual partnership and a difficulty in recognizing what she now considers to be abusive sexual encounters.

Alicia’s and Bethany’s stories could indicate a gendered dimension of PSSD; a study investigating differences in elicited cognitive appraisals during sexual activity found that women with SD report experiencing thoughts related to feeling abused, disrespected or violated during sexual activity significantly more often than sexually healthy women (Nobre & Pinto-Gouveia, 2008). Thus, whilst PSSD has a marked impact on the sexual experiences of both men and women, the nature of this impact may be experienced differently. This poses important implications for clinicians and researchers working with people with PSSD; exploring the relational and/or sexual beliefs and experiences of women living with PSSD, in a safe space where they can openly explore any relationship dynamics, would provide valuable information that ensures that individuals are safeguarded against any arising concerns regarding safety in relationships.

Participants spoke of significant loss as a result of PSSD, a condition that can fundamentally change one’s life entirely. For some, there was no reprieve from the enormity of what they faced on a daily basis. Others, however, were able to reflect on what has become important to their well-being. Talking therapy that is compassionate and validating, whilst being centred on the individual’s unique story, was helpful in some cases. Additionally, focusing on what one can control (e.g., diet and exercise), and having distractions and a routine can also be useful. Research has also shown that a sense of belonging is central to positive mental wellbeing (Allen et al., 2021), correlating with perceived meaningfulness of life (Lambert et al., 2013). It is thus no surprise that some participants spoke of how important the PSSD community had become in their lives, providing that sense of comradery and connection. Whilst it is recognized that it can be difficult to hear and/or read about others’ difficult experiences (necessitating boundaries around this), participants also felt that being part of a supportive network can provide a sense of feeling understood when others around you may be dismissive, an experience that people with PSSD know very well.

Whilst there is no doubt that PSSD is not a universal outcome of SSRI use, the body of literature demonstrating its prevalence and harm continues to grow. As mental health professionals working on an almost daily basis with individuals who could be at risk of PSSD, the experiences of participants interviewed for this research will often be at the forefront of our minds. Completing this work has highlighted the dichotomy that comes with being both a clinician, often working with individuals who consider their medication helpful, and a researcher, hearing the stories of people whose lives were changed by the medication for very different reasons. The use of supervision and reflexive journalling has been instrumental in ensuring that there was space to reflect on these issues thoughtfully, allowing us to complete our roles with the rigour and compassion that both patients and participants deserve.

The impact of invalidating experiences with professionals was evident in the participants’ interviews. It was necessarily transparent to participants that the lead researcher undertaking this research is a trainee clinical psychologist working in NHS settings. Whilst every effort was made to ensure that the interview could be a safe space for participants to share their stories (e.g., by sharing the interview guide ahead of time, ensuring participants knew that they could withdraw or decline any question at any time, etc), some participants may have understandably found it difficult to be fully open during our interviews. Given the sensitivity of the topics discussed in the interview, we were also mindful of the interviewer's identity as a male healthcare professional and how this may impact the recruitment of female participants. On reflection, ensuring that participants were aware of the questions in advance and that they could decline to answer and/or withdraw at any point was likely important for ensuring that they felt safe and able to be interviewed.

### Strengths and limitations

To our knowledge, this project constitutes the first qualitative study on PSSD in the UK, giving a voice to participants who have been living with PSSD—some for many years—and navigating NHS services with the condition. The participants’ accounts also provide a lens into the attitudes of local prescribers and other healthcare professionals towards PSSD, highlighting gaps in knowledge regarding the condition and/or willingness to engage in conversations about possible persistent side effects with patients. Recruiting ten participants also provided a rich data set and valuable insight into how PSSD impacts people’s lives. Importantly, participants were able to share deeply personal and meaningful reflections, which have contributed to our understanding of how SSRI-related sexual dysfunction affects identity, emotions, relationships, and experiences with professionals and therapy in the NHS.

There are several limitations to this study. Most participants identified as being of the same ethnic background (White British). Attitudes towards sex and sex-related problems can differ significantly across different cultures (Hall, 2019), and the inclusion of more participants from diverse backgrounds may have further enriched the findings of this study. That being said, given that generalizability is not the aim, homogeneous samples are typically considered helpful and appropriate for IPA research (Smith et al., 2022). Additionally, the majority of participants were single at the time of the interview, meaning that there were very few current accounts of the impact PSSD has on romantic relationships. However, this is likely reflective of the wider community, with recent research indicating that the majority of people living with PSSD identified as single (Linke et al., 2025).

### Clinical implications

This study poses several considerations for clinical practice, including for psychologists and therapists. Healthcare professionals, including prescribers such as GPs, should be mindful of the impact PSSD can have on patients in their care, as well as the distress caused both by persistent side effects and any automatic rejection of those side effects. It is clear from the participants’ interviews that an honest, transparent conversation regarding SSRI-related SD would have made a significant difference to their experience.

Talking therapy that is person-centred, compassionate and validating may be beneficial to some people with PSSD. Given the number of SSRI prescriptions filled each year in the UK, mental health professionals will frequently work with individuals experiencing SSRI-related SD. It is important that practitioners educate themselves on the nature of these side effects, the potential for these side effects to persist following SSRI use, and the impact this experience can have on individuals. Importantly, practitioners should be aware of the impact that PSSD can have on experiences of emotion and the implications this may have for the individual’s treatment goals and the appropriate intervention plan. Additionally, practitioners should be sensitive to possible vulnerabilities that can be caused by experiencing SD, ensuring that individuals have a safe space to discuss any concerns they may have regarding their sex lives or relationships.

### Recommendations for future research

Whilst the literature on PSSD is expanding, there is still a great need for more research to help us understand this condition. From a psychological perspective, further studies to help unpick individuals’ experiences of therapy, including people from diverse backgrounds or characteristics, could be useful in ascertaining what helpful interventions could precisely entail. Clinical trials of psychological interventions for individuals distressed by PSSD could also be highly beneficial for people living with this condition, with a potential focus on therapies designed to increase emotional awareness, allowing us to consider whether these interventions may be of any benefit. Moreover, to our knowledge, no research has investigated the beliefs and attitudes of prescribers and healthcare professionals in the UK regarding PSSD. This research could be impactful in identifying gaps in knowledge, barriers to discussing PSSD with patients, and thoughts or concerns regarding possible treatment options.

It is clear from participants’ accounts—both from our study and the wider literature—that finding effective medical interventions for PSSD would be life-changing. Whilst a small number of retrospective studies in male populations have shown some possible benefits to pharmacological or behavioural interventions (De Luca et al., 2022; Reisman et al., 2022), there remains a need for randomized controlled trials with larger sample sizes and, importantly, including female participants to evaluate the efficacy of any existing treatment options that may be considered by clinicians.

Various studies have utilized patient-reported outcome measures (PROMs) to assess psychotropic-related SD (Brugi et al., 2026), providing valuable information to clinicians on the extent and severity of sexual side effects. However, to our knowledge, no study has evaluated the use of PROMs for the assessment of PSSD, and further research could investigate whether existing measures can be used to provide insight into appropriate norms and cutoff scores/ranges for sexual functioning following SSRI cessation.

## Conclusion

This study investigated the lived experience and impact of PSSD for ten participants in the UK using an IPA methodology. The findings from this study highlight the distressing experiences that people with PSSD often have when raising their concerns in NHS settings, and the impact that this dismissal can have on their well-being and trust in healthcare services. Additionally, the participants spoke of the impact of PSSD on every aspect of daily life, from one’s personal identity and sense of self, to relationships with others, including friends, family and professionals. Mental health professionals can be helpful or harmful when interacting with people with PSSD, and this study identified clear recommendations—such as offering validation and being informed on the condition—for practitioners working with people who take SSRIs or other psychotropic medications. The participants’ accounts also highlighted strategies they utilized to manage their own well-being, using what they can to cope with the gravity of what they have experienced. Overall, this study offered an opportunity for participants to share their experiences of living with PSSD and, importantly, an opportunity for their stories to be heard.

## Data Availability

The data that support the findings of this study are available on request from the corresponding author. The data are not publicly available due to containing information that could compromise the privacy of research participants.
